# Heavier smoking increases coffee consumption: findings from a Mendelian randomization analysis

**DOI:** 10.1093/ije/dyx147

**Published:** 2017-08-14

**Authors:** Johan H Bjørngaard, Ask Tybjærg Nordestgaard, Amy E Taylor, Jorien L Treur, Maiken E Gabrielsen, Marcus R Munafò, Børge Grønne Nordestgaard, Bjørn Olav Åsvold, Pål Romundstad, George Davey Smith

**Affiliations:** dyx147-1 NTNU, Norwegian University of Science and Technology, Department of Public Health and General Practice, Trondheim, Norway,; dyx147-2 Forensic Department and Research Centre Brøset, St Olav's University Hospital Trondheim, Trondheim, Norway,; dyx147-3 Faculty of Health and Medical Sciences, University of Copenhagen, Copenhagen, Denmark,; dyx147-4 Department of Clinical Biochemistry and the Copenhagen General Population Study, Herlev and Gentofte Hospital, Copenhagen University Hospital, Herlev, Denmark,; dyx147-5 UK Centre for Tobacco and Alcohol Studies, School of Experimental Psychology, University of Bristol, Bristol, UK,; dyx147-6 MRC Integrative Epidemiology Unit (IEU) at the University of Bristol, Bristol, UK,; dyx147-7 Behavioural Science Institute, Radboud University, Nijmegen, The Netherlands,; dyx147-8 School of Social and Community Medicine, University of Bristol, Bristol, UK,; dyx147-9 NTNU, Norwegian University of Science and Technology, K.G. Jebsen Center for Genetic Epidemiology, Trondheim, Norway and; dyx147-10 Department of Endocrinology, St Olavs Hospital, Trondheim University Hospital, Trondheim, Norway

**Keywords:** Coffee, tea, smoking, Mendelian randomization

## Abstract

**Background:**

There is evidence for a positive relationship between cigarette and coffee consumption in smokers. Cigarette smoke increases metabolism of caffeine, so this may represent a causal effect of smoking on caffeine intake.

**Methods:**

We performed Mendelian randomization analyses in the UK Biobank (*N* = 114 029), the Norwegian HUNT study (*N* = 56 664) and the Copenhagen General Population Study (CGPS) (*N* = 78 650). We used the rs16969968 genetic variant as a proxy for smoking heaviness in all studies and rs4410790 and rs2472297 as proxies for coffee consumption in UK Biobank and CGPS. Analyses were conducted using linear regression and meta-analysed across studies.

**Results:**

Each additional cigarette per day consumed by current smokers was associated with higher coffee consumption (0.10 cups per day, 95% CI: 0.03, 0.17). There was weak evidence for an increase in tea consumption per additional cigarette smoked per day (0.04 cups per day, 95% CI: −0.002, 0.07). There was strong evidence that each additional copy of the minor allele of rs16969968 (which increases daily cigarette consumption) in current smokers was associated with higher coffee consumption (0.16 cups per day, 95% CI: 0.11, 0.20), but only weak evidence for an association with tea consumption (0.04 cups per day, 95% CI: -0.01, 0.09). There was no clear evidence that rs16969968 was associated with coffee or tea consumption in never or former smokers or that the coffee-related variants were associated with cigarette consumption.

**Conclusions:**

Higher cigarette consumption causally increases coffee intake. This is consistent with faster metabolism of caffeine by smokers, but could also reflect a behavioural effect of smoking on coffee drinking.

**Key Messages**

- Heavier smoking causes increased coffee consumption.
- This finding is strengthened by its consistency across three studies from the UK, Norway and Denmark with different patterns of coffee consumption.
- Evidence for a causal effect between smoking and tea consumption is weaker, possibly reflecting lower caffeine content in tea.
- There is no clear evidence that coffee consumption increases cigarette consumption but this may reflect lower power to detect an effect.

## Introduction

Smoking and coffee consumption have been shown to be strongly positively associated; smokers consume more coffee than non-smokers and, within smokers, smoking heaviness is associated with higher coffee consumption.^1^ Whether this relationship is specific to coffee, or extends to other caffeinated beverages, is less clear; however, a recent analysis in a UK cohort found a similar positive relationship with tea, suggesting it could apply more generally to caffeine.^2^

Given the known harmful effects of smoking and the widespread interest in the health effects of coffee or caffeine, which have been implicated (often as a protective factor) in a number of diseases including cancer, depression and diabetes,^3–7^ it is important to understand what drives this association. Cigarette smoking induces the cytochrome P450 1A2 (*CYP1A2*) enzyme, which is the primary enzyme involved in caffeine metabolism, and experimentally smoking decreases levels of plasma caffeine.^8–12^ Therefore, accelerated elimination of caffeine by tobacco smoking may lead to increased caffeine tolerance and coffee consumption.^13^ There may also be behavioural associations, as cigarettes and coffee are often consumed together. However, there are few data on whether smoking leads to increased coffee or caffeine consumption at the population level. Inferring causality from observational studies is difficult, due to the problems of confounding (by unmeasured or poorly measured factors) and reverse causality.

One potential way to minimize these problems is to use genetic variants which are associated with exposures as proxies for measured exposures in a Mendelian randomization analysis.^14^^,^^15^ A single nucleotide polymorphism, rs16969968 in the *CHRNA5* nicotinic receptor subunit gene, influences smoking heaviness (or tobacco consumption) within smokers.^16^ Each additional copy of the minor allele of rs16969968 (or its proxy, rs1051730) is robustly associated with smoking, on average, one additional cigarette per day.^17^^,^^18^ Genetic variants have also been identified which robustly associate with coffee consumption, with the two most strongly associated loci located between the *CYP1A1/2* genes (e.g. rs2472297) and near the aryl hydrocarbon receptor (*AHR*) gene (e.g. rs4410790).^19–21^

Of note, the smoking heaviness variant was reported to be associated with coffee consumption in a coffee consumption genome-wide association study (GWAS), but at a nominal level of significance and in a sample combining both smokers and non-smokers.^22^ A recent Mendelian randomization analysis of smoking and caffeine in a UK study and in a Dutch study (which did stratify into current, former and never smokers) did not find any evidence for a causal effect of smoking on caffeine intake or for a causal effect of caffeine intake on smoking.^23^ However, the sample sizes used were modest, so it is likely that these analyses were underpowered. A recent analysis of the association of coffee-related genetic variants with daily cigarette consumption including data from UK Biobank did not support a causal effect of coffee consumption on increased cigarette consumption.^24^ We sought to extend this work by performing a Mendelian randomization analysis to investigate whether there is a causal effect of smoking on coffee and tea consumption, in three large studies: the UK Biobank, the Norwegian HUNT study and the Copenhagen General Population Study (CGPS). In addition, we performed a Mendelian randomization analysis to investigate the causal effect of coffee consumption on cigarette consumption in the UK Biobank and the CGPS.

## Methods

### Study populations

We included individuals from three studies: the UK Biobank, which recruited over 500 000 men and women (aged 37 to 73 years) between 2006 and 2010,^25^ the second wave of the Norwegian HUNT study (1995–97),^26^ which invited all adults aged 20 years and older in the county of Nord-Trøndelag to participate, and the Copenhagen General Population Study (CGPS), a prospective cohort study with ongoing enrolment started in 2003.^27^ Full details of the study populations, including participation flowcharts, are available in Supplementary material (Supplementary Figures S1–S3, available as Supplementary data at *IJE* online).

### Genotyping

Information on genotyping of rs16969968 (UK Biobank) and rs1051730 (HUNT and CGPS) and on rs4410790 and rs2472297 (in UK Biobank and the CGPS) is provided in Supplementary material, available at *IJE* online. Coffee-related single nucleotide polymorphisms (SNPs) were not available in the HUNT study.

### Smoking behaviour

In all studies, smoking status was classified as never smoker, former smoker, current smoker and ever smoker (current and former smokers combined). Current smokers were also asked how many cigarettes they smoked per day. Full details of how the smoking variables were derived in each study are available in Supplementary material, available at *IJE* online.

### Coffee and tea consumption

Coffee and tea consumption were self-reported as the number of cups consumed daily. Full details of how these variables were collected in the individual studies are available in Supplementary material at *IJE* online. Information on whether type of coffee consumption was most commonly caffeinated or decaffeinated was available in UK Biobank, but not in HUNT or Copenhagen. However, consumption of decaffeinated coffee in Norway and Denmark is low.^28^^,^^29^ Information on whether tea consumption was caffeinated or decaffeinated was not available in any of the studies.

### Covariates

Observational analyses were adjusted for age, sex and educational attainment. A full description of the covariates used in each study is provided in Supplementary material, available at *IJE* online.

### Statistical analysis

Analyses were conducted in Stata (version 14. 1). Within each study, associations between smoking status (never, former and current) and smoking heaviness (cigarettes per day) and continuous measures of coffee and tea were investigated using linear regression. These analyses were adjusted for age, sex and educational attainment. Robust standard errors were used to account for non-normality of residuals, as tea and coffee data tended to be right skewed.

We assessed the association between the genotypes (rs16969968/rs1051730, coded as 0,1,2 according to the number of smoking heaviness-increasing alleles) and smoking heaviness using linear regression. In UK Biobank and the CGPS, we created an unweighted genetic risk score from the two coffee-related SNPs, coded as 0,1,2,3 or 4, according to the number of coffee consumption-increasing alleles (major allele for rs4410790 and minor allele for rs2472297). We assessed the association of this risk score with coffee consumption in daily smokers using linear regression.

In Mendelian randomization analysis, we used linear regression to investigate associations of the smoking heaviness-related variants with amount of coffee and tea consumption, and logistic regression to investigate associations with any compared with no consumption of these drinks. These analyses were performed stratified by smoking status (never, former, current and ever). As the genetic variant is only associated with smoking heaviness in individuals who smoke, we should only see evidence for an association with coffee or tea in current smokers (and perhaps former smokers if the effects are long-lasting). An association with coffee or tea consumption in never smokers would suggest a pleiotropic effect of the variant (not acting through cigarette consumption). Associations of the coffee genetic risk score with daily cigarette consumption were investigated using linear regression. Genetic analyses were adjusted for age, sex (in all studies) and principal genetic components (in UK Biobank only).

All analyses were performed within individual studies and combined in inverse variance weighted fixed-effect meta-analyses. We assessed heterogeneity between associations in the different smoking categories using Cochran’s Q and the I-squared statistic. When there was evidence of heterogeneity (I^2 ^> 50%), we used a random-effects meta-analysis. Primary analyses were performed only in individuals who reported consuming coffee or tea, but we also conducted sensitivity analyses including all individuals (consumers and non-consumers).

## Results

The analysis sample consisted of 114 029 individuals from UK Biobank, 56 664 individuals from the HUNT study and 78 650 individuals from the Copenhagen General Population Study (see Table 1). Smoking prevalence was higher in the HUNT study than in CGPS and UK Biobank. Coffee consumption was more common in the HUNT and CGPS than in UK Biobank, but tea consumption was more common in the UK Biobank than in HUNT and CGPS. Among consumers, individuals from the UK Biobank tended to drink more cups of tea per day, but less cups of coffee per day, than in HUNT and CGPS. In all studies, there was a negative correlation between coffee and tea consumption when considering individuals who consumed at least some tea or coffee (Supplementary Table S1, available as Supplementary data at *IJE* online). However, correlations were weaker in individuals reporting both tea and coffee consumption.

**Table 1:** Characteristics of the study populations

	UK Biobank (114029)^b^			HUNT (56664)^b^			CGPS (78650)^b^		
	Mean/*N*	SD/IQR/%	Range	Mean/*N*	SD/IQR/%	Range	Mean/*N*	SD/IQR/%	Range
Age	56.9	7.9	40–73	49.9	17.1	19–101	57.6	13.1	20–99
Sex									
Male	53893	47.3		27021	47.7		35288	55.1	
Female	60136	52.7		29643	52.3		43362	44.9	
Educational attainment^a^									
Low	20501	18.1		19759	36.6		17560	22.4	
Middle	40829	36.1		23514	43.6		27456	35.0	
High	51687	45.7		10653	19.8		33376	42.6	
Smoking status									
Never	61179	53.7		24747	43.7		32168	40.9	
Former	39006	34.2		14350	25.3		31553	40.1	
Current	13844	12.1		17528	31.0		14929	19.0	
Cigarettes per day current smokers	17.0	8.2		11.2	5.7		15.4	9.1	
Tea/coffee consumption									
Any tea consumption	96522	84.7		18130	55.7		45789	58.2	
Any coffee consumption	89789	78.7		49991	91.0		70228	89.3	
Any tea or coffee consumption	111582	97.9		52512	95.0		77023	97.9	
Tea consumption (cups per day) in tea drinkers: median (IQR)	4	2,5	0.5–25	2	1,2	1–21	1.1	0.6, 2.3	0.1–25
Coffee (cups per day) in coffee drinkers: median (IQR)	2	1,4	0.5–25	5	2,5	1–50	2.9	1.4, 4	0.1–25

^a^Education defined as follows. UK Biobank: high: degree/professional qualifications, middle: school/vocational qualifications, low: no qualifications. HUNT: high: college/university education (> 12 years), middle: secondary education (11–12 years), low: primary education (< 10 years). CGPS: High: university degree, Middle: college degree, Low: no education/studying/vocational qualifications/short education.

^b^Number of participants with genotype information, varies according to missing values on different variables.

### Associations of tea and coffee consumption and smoking with demographic factors

Similar patterns of smoking with age, sex and education were observed across the studies (see Supplementary Tables S2–S4, available as Supplementary data at *IJE* online). However, there were some differences in patterns of coffee and tea consumption by demographic factors between the studies (see Supplementary Tables S5–S7, available as Supplementary data at *IJE* online).

### Observational associations between smoking and coffee and tea consumption

**Figure 1:**
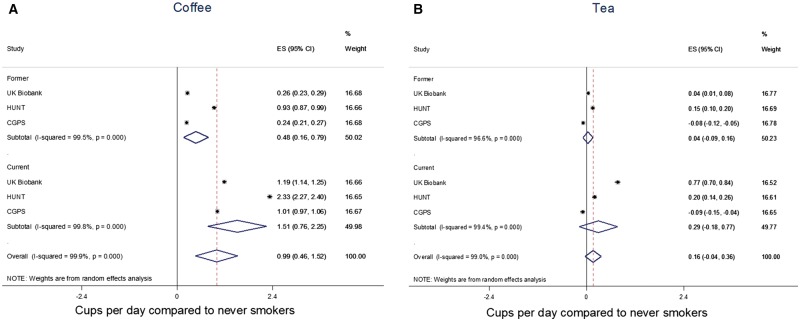
Associations between smoking status and tea and coffee consumption. Beta coefficients represent difference in coffee/tea consumption in former and current compared with never smokers. Analyses adjusted for age, sex and educational attainment. From linear regression using robust standard errors to account for non-normality of residuals. Estimates combined in a random-effects meta-analysis.

**Figure 2:**
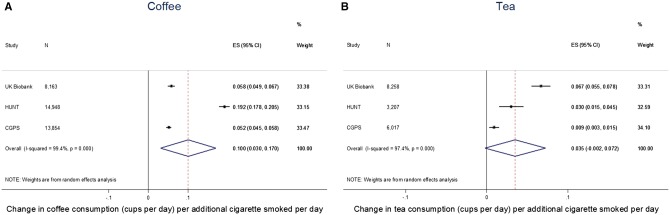
Associations between number of cigarettes per day among current smokers and coffee and tea consumption. Beta coefficients represent difference in coffee/tea consumption in current smokers per additional cigarette consumed per day. Analyses adjusted for age, sex and educational attainment. From linear regression using robust standard errors to account for non-normality of residuals. In UK Biobank, *N* represents number of current daily smokers only. Estimates combined in a random-effects meta-analysis.

In observational analyses of smoking status and smoking heaviness (cigarettes per day) with coffee and tea consumption, there was a high degree of heterogeneity between studies (I^2 ^> 95%). Former and current smoking were associated with higher coffee consumption (Figure 1), but there was no overall evidence from the meta-analysis that smoking status was associated with tea consumption. Among smokers, each additional cigarette smoked per day was positively associated with both coffee and tea consumption, although the association was larger for coffee (0.10 cups per day, 95% CI: 0.03, 0.17) than for tea (0.04 cups per day, 95% CI: -0.002, 0.07) (Figure 2). Mutual adjustment of associations for tea and coffee consumption did not make any material difference to results (see Supplementary Tables S8 and S9, available as Supplementary data at *IJE* online).

### Mendelian randomization: smoking heaviness to coffee and tea consumption

In the combined estimate, each additional copy of the minor allele of rs16969968/rs1051730 was associated with smoking 0.83 additional cigarettes per day (95% CI: 0.63, 1.03, I^2^ 71%, combined using random-effects meta-analysis, Supplementary Table S10, available as Supplementary data at *IJE* online). There was no clear consistent evidence that rs16969968/rs1051730 was associated with sex or education in any of the studies. When stratified by smoking status, there was some evidence in the UK Biobank that rs16969968/rs1051730 genotype was associated with age (age decreased per copy of the minor allele) in former and current smokers but not never smokers (see Supplementary Tables S11–S22, available as Supplementary data at *IJE* online). This is consistent with the known effects of this variant on mortality.^30^

In the combined estimate from the meta-analysis, rs16969968/rs1051730 genotype was not associated with consuming any coffee compared with no coffee, nor with consuming any coffee or tea compared with no coffee or tea (see Supplementary Figures S4–S5, available as Supplementary data at *IJE* online). However, the minor allele was associated with reduced odds of consuming any tea compared with no tea consumption (OR: 0.94, 95% CI: 0.91, 0.97) (see Supplementary Figure S6, available as Supplementary data at *IJE* online).

**Figure 3:**
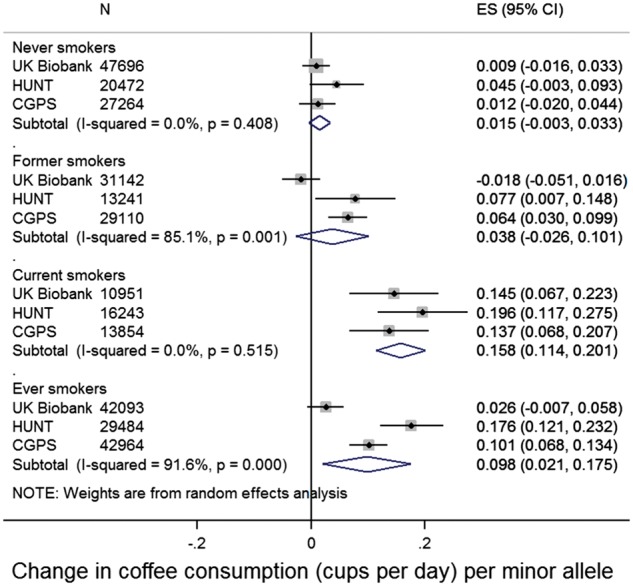
Associations between rs16969968/rs1051730 and coffee consumption. Adjusted for age, sex (in all studies) and principal components (in UK Biobank). Analyses restricted to individuals reporting at least some coffee consumption. In UK Biobank, ‘current smokers’ includes daily and occasional current smokers. Estimates combined in a random-effects meta-analysis.

There was strong evidence that the minor allele of rs16969968/rs1051730 was associated with increased coffee consumption in current smokers (Figure 3). As there was evidence for heterogeneity between studies in some smoking groups (former and ever smokers), results from random effects meta-analyses are presented. Each additional copy of the minor allele was associated with a 0.16-cup increase in coffee consumption (95% CI: 0.11, 0.20). There was no clear evidence that rs16969968/rs1051730 was associated with coffee consumption in never smokers (beta 0.02, 95% CI: -0.003, 0.03) or in former smokers (beta 0.04, 95% CI: -0.03, 0.10). There was evidence for heterogeneity between studies in the estimate in former smokers (I^2^ 85%). Including non-consumers of coffee in this analysis did not materially change results (Supplementary Figure S7, available as Supplementary data at *IJE* online).

**Figure 4:**
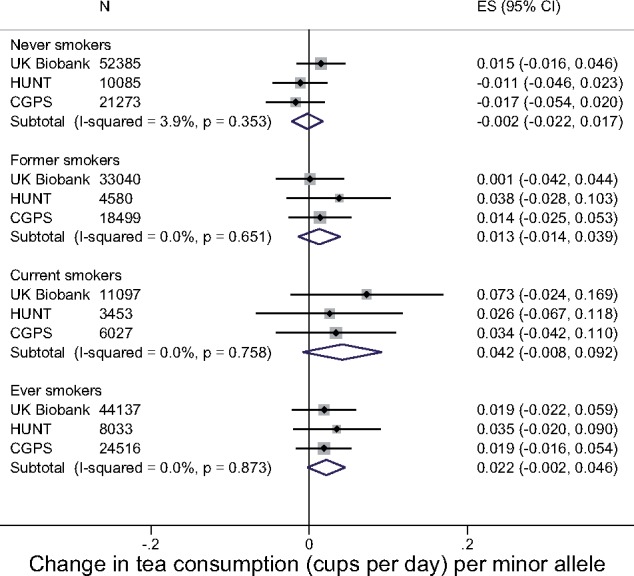
Associations between rs16969968/rs1051730 and tea consumption. Adjusted for age, sex (in all studies) and principal components (in UK Biobank). Analyses restricted to individuals reporting at least some tea consumption. In UK Biobank, ‘current smokers’ includes daily and occasional current smokers. Estimates combined in a fixed-effects meta-analysis.

There was no strong evidence for an association between rs16969968 and tea consumption in any of the smoking categories (Figure 4). The association was strongest in current smokers [0.04 cups per day per additional minor allele (95% CI: −0.01, 0.09)]. However, there was no clear statistical evidence for differences in the association between smoking categories (*P*_heterogeneity_ = 0.22). Including non-consumers of tea in this analysis weakened the magnitude of association seen in both current and former smokers (Supplementary Figure S8, available as Supplementary data at *IJE* online).

Given the negative correlation between coffee and tea consumption, we also performed analyses combining coffee and tea consumption (by adding together cups of tea and coffee consumed). These results were similar to those observed for coffee consumption alone (see Supplementary Figure S9, available as Supplementary data at *IJE* online). Among individuals consuming some tea or coffee, there was evidence that the minor allele was associated with an increase in the ratio of coffee to tea consumed among ever smokers (beta 0.04, 95% CI: 0.01, 0.07) but not among never smokers (beta -0.002, 95% CI: −0.02, 0.02) (Supplementary Figure S10, available as Supplementary data at *IJE* online). Similarly, among individuals consuming different amounts of coffee and tea, the minor allele was associated with increased odds of drinking more coffee than tea in current [odds ratio (OR) per minor allele: 1.06, 95% CI: 1.01, 1.10] and ever smokers (OR per minor allele: 1.02, 95% CI: 1.00, 1.05) but not in never smokers (OR per minor allele: 0.99, 95% CI: 0.97, 1.01) (Supplementary Figure S11, available as Supplementary data at *IJE* online).

Within UK Biobank, data were available on type of coffee most commonly consumed. A similar pattern of results was obtained for both caffeinated and decaffeinated consumption, although confidence intervals for decaffeinated coffee consumption were wide (see Supplementary Figure S12, available as Supplementary data at *IJE* online). To test whether associations were limited to caffeinated beverages or reflected a general effect of smoking heaviness on thirst, we also performed a Mendelian randomization analysis in UK Biobank with water consumption as the outcome (Supplementary Figure S13, available as Supplementary data at *IJE* online). There was no clear evidence that rs16969968/rs1051730 was associated with water consumption in any of the smoking categories, although the minor allele was associated with lower water consumption among current smokers, but the precision of this estimate was low.

### Mendelian randomization: coffee consumption to smoking heaviness

In current smokers from UK Biobank (*N* = 10 665) and CGPS (*N* = 14 929), each coffee consumption-increasing allele of the genetic risk score was associated with consuming 0.09 additional cups of coffee per day (95% CI: 0.07, 0.12) (Supplementary Table S23, available as Supplementary data at *IJE* online). There was no clear evidence for an association between the coffee genetic risk score and cigarette consumption (age- and sex-adjusted beta per coffee consumption-increasing allele: -0.01 cigarettes per day, 95% CI: -0.07, 0.05). This association was similar in coffee consumers and non-consumers (Supplementary Table S24, available as Supplementary data at *IJE* online).

## Discussion

In three large studies from the UK, Norway and Denmark, which have different patterns of coffee and tea consumption, we have demonstrated that being a smoker is associated with higher coffee intake, and that within smokers, tobacco consumption is positively associated with coffee consumption. The results from our Mendelian randomization analysis of coffee consumption were largely consistent across studies and provide evidence that heavier smoking causally increases coffee intake. Although the pattern of results in the Mendelian randomization analysis was similar for tea consumption, we did not find clear evidence for a causal effect of smoking heaviness on tea consumption. In addition, we did not find evidence to support a causal effect of coffee intake on daily cigarette consumption in current smokers, suggesting that this may not be a bidirectional effect.

Our findings from the Mendelian randomization analysis, that the smoking-increasing allele of rs16969968/rs1051730 is positively associated with coffee intake in current smokers, suggest that a causal effect of smoking on coffee consumption could explain a large part of the observational association between smoking heaviness and coffee consumption. The magnitude of the association in the genetic analysis (0.16 additional cups of coffee per minor allele) is similar, and in fact slightly larger than the observational association, given that the minor allele of this variant is associated with smoking around one additional cigarette per day.^17^ This may reflect measurement error in the assessment of cigarettes per day, or the fact that rs16969968 is a marker of total tobacco consumption which is likely to vary even between individuals smoking the same number of cigarettes, due to differences in the way individuals smoke their cigarettes (e.g volume of smoke inhaled).^18^^,^^31^ It is for this reason that we did not perform a formal instrumental variable analysis of the effect of cigarettes smoked per day and coffee and tea consumption. As discussed previously in relation to Mendelian randomization studies of tobacco, use of a measured exposure which does not fully capture the exposure determined by the genetic variant is a violation of the exclusion restriction assumption of instrumental variable analysis, and can lead to biased estimates of the magnitude of effects.^32^^,^^33^ To put the magnitude of the smoking heaviness effect reported here into context, the size of the association between rs16969968 and coffee consumption in current smokers is similar to that observed for the most strongly associated SNPs identified in GWAS of coffee consumption.^19^^,^^21^^,^^34^

Lack of association of rs16969968/rs1051730 with coffee consumption in never smokers provides us with some evidence that this association is not due to pleiotropic effects of the variant (i.e. associations with the outcome not via the exposure of interest). Furthermore, this finding is strengthened by its consistency across studies from countries with different patterns of coffee consumption. The small positive association observed in former smokers in HUNT and CGPS suggests that there may be some residual effect that persists following smoking cessation. The reason for a lack of association in former smokers in UK Biobank is unclear; it is possible that time since quitting in UK Biobank is longer than in the other studies, as the sample is restricted to older individuals. We also repeated analyses in the UK Biobank excluding occasional past smokers (as UK Biobank has a high proportion of occasional smokers) but this did not substantially alter the association.

There are two key mechanisms through which smoking may increase coffee consumption. First, it is well known that cigarette smoke increases activity of the caffeine metabolizing enzyme CYP1A2*.* Polycyclic aromatic hydrocarbons in tobacco smoke bind to the aryl hydrocarbon receptor, which activates the *CYP1A2* gene.^8^ Faster metabolism of caffeine in smokers^35^^,^^36^ would require them to consume more caffeine to experience its stimulating effects and avoid withdrawal, but would also allow them to consume higher levels before experiencing symptoms of caffeine toxicity.^37^ Second, the association may be due to behavioural links between smoking and coffee consumption, with smoking acting as a cue or providing an opportunity (i.e. a cigarette break) to consume coffee.^1^ The association of rs16969968 with consumption of decaffeinated coffee in current smokers in UK Biobank offers some support for this behavioural explanation. It is likely that decaffeinated coffee consumers were once caffeinated coffee consumers, so this could just reflect continuation of an established behaviour. However, as UK Biobank participants were only able to indicate their main type of coffee consumption in the questionnaire, it is likely that at least some of the coffee consumption in this ‘decaffeinated’ analysis is caffeinated. Furthermore, the lack of clear evidence for an association with tea suggests that the coffee-smoking association is unlikely to be entirely behavioural.

Given the effect of tobacco smoke on caffeine metabolism, it is perhaps surprising that we did not find clear evidence for an association with tea consumption. It is possible that the effect of smoking is limited to coffee; the literature on the association between smoking and tea drinking is less consistent than with coffee drinking, with some studies finding positive^2^ and others null or negative associations.^1^^,^^2^^,^^38^ However, this could simply reflect cultural differences in tea consumption between the countries in this analysis; in UK Biobank, which has the highest tea consumption of all the studies, there was suggestive evidence for a causal effect of smoking on tea consumption. Interestingly, the effect size in UK Biobank was around half of that observed for coffee, which might be expected given the lower caffeine content of tea (typically around half that of coffee).^2^ We were also unable to distinguish between caffeinated and decaffeinated tea consumption in any of the studies; if the association is due to effects of smoking on caffeine metabolism, including decaffeinated tea would have weakened associations. Heavier smoking may cause individuals to preferentially drink coffee rather than tea due to its higher caffeine content.^39^ Analyses of coffee and tea preference (Supplementary Figures 8 and 9), and the negative association between the minor allele of rs16969968/rs1051730 and consumption of any tea, suggest that this might be the case. Therefore, lack of association with tea consumption in this analysis may reflect lower statistical power to detect smaller associations with tea, cultural differences in tea consumption, or masking of associations by preference for coffee.

Our analysis, along with previously published analyses including GWAS data and data from the UK Biobank,^24^ did not find evidence that coffee consumption causally increases smoking heaviness among smokers. The rs4410790 and rs2472297 SNPs are in or near genes involved in caffeine metabolism (*CYP1A2* and *AHR*) and have also been shown to associate with tea consumption.^40^ Therefore, lack of association can be taken more broadly as evidence that increased caffeine consumption may not cause increased cigarette consumption. However, it is likely that this analysis was underpowered, so we cannot rule out the possibility of a causal effect in this direction.

There are several limitations to this analysis. First, the UK Biobank had a response rate of around 5% and is not very representative of individuals of this age group in the UK. Our results could be affected by collider bias if selection into the sample is related to both coffee consumption and smoking.^41^ However, the consistency of the result for coffee consumption with that seen in HUNT and CGPS, which have higher response rates and are more likely to be representative of the general population, suggest that this is unlikely to be a major source of bias. Additionally, the UK Biobank initial GWAS release contains about 50 000 individuals selected on the basis of smoking status and smoking heaviness.^42^ However, exclusion of these individuals from the UK Biobank analysis did not materially change results (see Supplementary Figure S14). We also cannot rule out the possibility that stratification by smoking status might induce collider bias if the genetic variant itself determines smoking status.^43^ Although rs16969968/rs1051730 is largely a determinant of smoking heaviness within smokers^17^ and does not appear to influence smoking initiation, there is evidence that the minor allele is associated with being a current compared with a former smoker.^44^

Tea and coffee consumption were self-reported and it is likely that cup size and strength of coffee and tea differed between studies. We were also unable to evaluate associations with other potential sources of caffeine such as cola or chocolate, because they were not available in all studies. Therefore, magnitudes of effect should be treated with caution, particularly if being used to make inferences about the effect of smoking on caffeine consumption.

Misclassification of smoking status could be another potential source of bias, but should serve to make estimates of the association between rs16969968 and coffee consumption in never, former and current smokers more similar to each other.

Last, we have only evaluated smoking heaviness in this manuscript. The key genetic instrument for smoking initiation in the *BDNF* gene is likely to be pleiotropic as it also associates with other phenotypes, including coffee consumption, at a genome-wide significance level.^34^

In conclusion, we provide evidence that higher cigarette consumption increases coffee consumption, but not for a causal effect in the opposite direction. These findings confirm the need to consider both coffee and smoking together when considering the health implications of coffee or caffeine in population studies. In addition, given that it has been hypothesized that overlap in symptoms between caffeine toxicity and nicotine withdrawal could increase risk of relapse to smoking,^45^ our findings may have implications for smoking cessation if individuals trying to quit smoking experience caffeine toxicity due to reduced caffeine metabolism.

## Supplementary Data

Supplementary data are available at *IJE* online.

## Funding

CGPS: this work was supported by Herlev and Gentofte Hospital, Copenhagen University hospital. The funding organization had no role in the design and conduct of the study, the collection, analysis, and interpretation of the data, or in the writing of the paper. UK Biobank: this research has been conducted using the UK Biobank Resource (application 9142). A.E.T. and M.R.M. are members of the UK Centre for Tobacco and Alcohol Studies, a UKCRC Public Health Research: Centre of Excellence. Funding from British Heart Foundation, Cancer Research UK, Economic and Social Research Council, Medical Research Council and the National Institute for Health Research, under the auspices of the UK Clinical Research Collaboration, is gratefully acknowledged. This work was supported by the Medical Research Council (grant number: MC_UU_12013/1, MC_UU_12013/6). The Nord-Trøndelag Health Study (the HUNT Study) is collaboration between HUNT Research Centre (Faculty of Medicine, NTNU, Norwegian University of Science and Technology), Nord-Trøndelag County Council and the Norwegian Institute of Public Health.

**Conflict of interest:** None declared.

## Supplementary Material

Supplementary Data
