# Supplementary material for: Heavier smoking increases coffee consumption: findings from a Mendelian randomization analysis
Source: Int J Epidemiol. 2017 Aug 14;46(6):1958–67. doi: 10.1093/ije/dyx147 (PMC5837196; doi:10.1093/ije/dyx147)
Supplement: Supplementary Data [file supplementary_material_dyx147.docx]

**Additional methods**

**UK Biobank**

**Study population**

The UK Biobank ([www.ukbiobank.ac.uk](http://www.ukbiobank.ac.uk)) recruited over 500,000 men and women (aged 37 to 73 years) between 2006 and 2010 (1). Participants attended one of the 21 assessment centres in England, Wales and Scotland, where they provided information on demographic, lifestyle factors and medical history through interviews and questionnaires and had physical measurements and blood, urine and saliva samples taken. The full protocol for the study is available online (www.ukbiobank.ac.uk/wp-content/uploads/2011/11/UK-Biobank-Protocol.pdf). The UK Biobank study was approved by the North West Multi-Centre Research Ethics Committee and all participants provided written informed consent to participate in the UK Biobank study.

**Genotyping**

DNA was extracted from blood samples using the Promega Maxwell 16 Blood DNA Purification Kit (AS1010), which uses magnetic bead technology to purify the DNA. An initial sample of 152,249 individuals were genotyped for 641,018 SNPs, some using the Affymetrix UK BiLEVE Axiom array and some using the Affymetrix UK Biobank Axiom array. These two arrays are very similar, with an overlap of around 95%. Imputation was conducted using the 1000 genomes Phase 3 and UK10k reference panels. After quality control, just over 73 million SNPs were available for analysis. The rs16969968, rs4410790 and rs2472297 polymorphisms were imputed with info scores of 1. Amongst individuals of European ancestry, there was some evidence for deviation from Hardy Weinberg equilibrium for rs16969968 (p=0.02) but not for rs4410790 and rs2472297 (p≥0.39). This is likely due to the selection of participants on the basis of smoking in the UK BiLEVE sample (2). The analysis sample was restricted to unrelated individuals, based on a threshold of 0.05 estimated from genetic kinships, and to individuals of Caucasian genetic ancestry using principal components analyses (PCA). Individuals failing genotype quality control (N=180) were also excluded from analyses. This resulted in a sample of 114,478 individuals with genetic data and 114,029 individuals with both genetic data and information on tobacco and coffee and tea consumption.

**Smoking behaviour**

Participants were asked about current and past tobacco (cigarette, pipe, cigar or other) smoking behaviour in a computerized questionnaire. A full list of the questions is available at: <http://biobank.ctsu.ox.ac.uk/crystal/docs/TouchscreenQuestionsMainFinal.pdf>. The following questions were asked about current and past smoking status: “Do you smoke tobacco now?” (Yes, on most or all days, Only occasionally, No, Prefer not to answer) and “In the past, how often have you smoked tobacco?” (Smoked on most or all days, Smoked occasionally, Just tried once or twice, I have never smoked, Prefer not to answer). Individuals who indicated that they had tried tobacco but were not past or current daily or near daily smokers were also asked “Have you smoked more than 100 cigarettes in your lifetime?”. From these questions, the following smoking status categories were defined: never smokers (individuals who had smoked less than 100 cigarettes in a lifetime), former smokers (who had smoked more than 100 cigarettes but were not current smokers) and current smokers. Where individuals did not know or did not say whether they had consumed more than 100 cigarettes in their lifetime, those who said they had tried one or two in the past were classified as never smokers and those who said they smoked occasionally in the past as former smokers). Current regular smokers were asked about number of cigarettes consumed per day; answers were provided on a continuous scale. Pack years were calculated from reported daily cigarette consumption, age of starting smoking and age of stopping smoking (in former cigarette smokers) using the formula: pack years = (daily cigarette consumption/20) × years smoking. Years smoking was calculated as current age - age of starting in current smokers, and age of stopping - age of starting in former smokers. Where individuals reported starting smoking prior to age 16, pack years was calculated from age 16, as has been done in previous analyses of UK Biobank data (2). Where individuals reported starting and stopping smoking at the same age, it was assumed that they had smoked for 0.5 years.

**Coffee and tea consumption**

All participants were asked how many cups of tea (including black and green tea) and coffee (including decaffeinated coffee) they consumed each day. Answers were provided on a continuous scale. Individuals who said that they drank coffee but drank less than 1 cup of coffee a day were assigned a value of 0.5 and individuals reporting drinking more than 25 cups a day of either tea or coffee (N=406) were excluded from analyses. Coffee consumers were asked what type of coffee they usually drank and were able to select only one of the following options: decaffeinated, instant, ground, other, do not know, prefer not to say. Participants were not specifically asked which type of tea they drank. From the answers to these questions, daily caffeine consumption was calculated by multiplying the number of cups of tea and coffee consumed each day by the caffeine content of each drink.

**Covariates**

Age was coded as both a continuous and a categorical variable (38-44, 45-49, 50-54, 55-59, 60-64, 65+ years) in order to investigate linearity with tea and coffee consumption and cigarette smoking. Information educational attainment was self-reported in the main UK Biobank questionnaire. Individuals were asked to record all types of educational qualification that they had been awarded (College or University Degree, A-levels/AS-levels or equivalent (exams taken at age 18 years), O levels of equivalent (exams taken at age 16 years), CSEs or equivalent (exams taken at age 16 years), NVQ or HND or HNC or equivalent (vocational qualifications), Other professional qualifications, None of the above). For these analyses, this information was recoded into a three level variable: none, degree/professional and all other educational qualifications.

**HUNT**

**Study Population**

The second wave of the HUNT Study in Norway took place in 1995-97 (www.ntnu.edu/hunt). In the county of Nord-Trøndelag, all adults aged 20 years and older were invited to participate in the study. A total of 65,215 (69%) accepted the invitation and gave written informed consent to use the data for medical research. The data collection included questionnaires, clinical measurements and blood samples, and has been described in detail elsewhere (3). The present study was approved by the Regional Committee for Medical Research Ethics.

**Genotyping**

DNA has been extracted from blood samples for all participants of the HUNT 2 study and is stored at the HUNT biobank, as previously described (4). The rs1051730 polymorphism was genotyped at the HUNT biobank using TaqMan genotyping assays (Applied Biosystems Inc., Foster City, CA, USA) and performed on an Applied Biosystems 7900HT Fast Real-Time PCR System using 10 ng of genomic DNA, as previously described. The call rate cut-off was set to 90%, and the genotype frequencies were in agreement with HapMap data. The genotype was coded according to number of the minor T-allele, assuming an additive genetic model (0=no T allele, 1=heterozygote for T allele and 2=homozygote for T). Although there were no genetic measures, at the time of the survey (1995-97), the participants of the HUNT Study had a very low ethnic diversity (less than 3% non-European)(3). Genotype distributions did not differ from Hardy-Weinberg equilibrium (p=0.24). Altogether 56,664 participants were genotyped for rs1051730, and of these participants, 54,895 (84%) had information on tobacco and coffee consumption (Figure S2 supplementary material).

**Smoking behaviour**

The participants reported current and past tobacco (cigarette, pipe, cigar or other) smoking behaviour in a questionnaire available at: http://www.ntnu.edu/hunt/data/que. In the following study, the participants were classified as current, former, and never smokers based on the following questions: “Do you smoke?” ([Yes, cigarettes daily](https://hunt-db.medisin.ntnu.no/hunt-db/#variab3024), [Yes, cigar/cigarillos daily](https://hunt-db.medisin.ntnu.no/hunt-db/#variab3025), [Yes, pipe daily](https://hunt-db.medisin.ntnu.no/hunt-db/#variab3026) or [Never smoked daily](https://hunt-db.medisin.ntnu.no/hunt-db/#variab3027)). Previous smoking was assessed with multiple questions: “If ever smoked daily previously. [How long has it been since you stopped (Number of years)](https://hunt-db.medisin.ntnu.no/hunt-db/#variab3028)?”. And “If ever smoked daily previously. How long has it been since you stopped (Number of years)?”. Number of cigarettes daily was assessed with the question: “If currently or previously daily smoker. [How many cigarettes do you or did you usually smoke daily?](https://hunt-db.medisin.ntnu.no/hunt-db/#variab3029)” Pack years were calculated from info on years of daily smoking times number of packs smoked per day (with 20 cigarettes in a pack). This formula makes no attempt to compensate for inconsistent base variables.

**Coffee and tea consumption**

Coffee and tea consumption was self-reported as the number of cups daily based on the following questions: “How many cups of coffee/tea do you drink daily ([Brewed coffee?](https://hunt-db.medisin.ntnu.no/hunt-db/#variab3032), [Other types of coffee?](https://hunt-db.medisin.ntnu.no/hunt-db/#variab3033), Tea?)?” The answers were provided on a continuous scale.

**Covariates**

Age at participation was coded as both a continuous and a categorical variable (38-44, 45-49, 50-54, 55-59, 60-64, 65+ years). Information educational attainment was self-reported based on the following question: “What is your highest level of education (Primary school 7-10 years, continuation school, folk high school, High school, intermediate school, vocational school, 1-2 years high school, University qualifying examination, junior college, A levels, University or other post-secondary education, less than 4 years or University/college, 4 years or more)? For these analyses, this information was recoded into a three level variable: High: college/university education (>12 years), middle: secondary education (11-12 years), or low: primary education (<10 years).

**Copenhagen General Population Study**

**Study population**

The Copenhagen General Population Study (CGPS) is a prospective cohort study with ongoing enrolment started in 2003 (5, 6). At examination, all participants filled out a questionnaire, which was reviewed by an investigator at the attendance, and had a physical examination performed and blood samples taken for biochemical analyses and DNA extraction. The study was approved by Herlev and Gentofte Hospital and by Danish ethical committees, and was conducted according to the Declaration of Helsinki. Written informed consent was obtained from all participants.

**Genotyping**

Participants were genotyped for the rs1051730 polymorphism using *TaqMan*-based assays (Applied Biosystems Inc., Foster City, California) and for rs4410790 and rs2472297 polymorphisms near *AHR* and *CYP1A1/2* genes. Analyses included positive controls for each genotype verified by sequencing. Due to reruns, call rates were ≥99.7%. Genotype distributions did not differ from Hardy-Weinberg equilibrium. All individuals were white of Danish descent according to the Danish Central Person Registry. We included 78,650 individuals from the CGPS examined in 2003-2013, all with information on rs1051730, rs4410790, and rs2472297 genotype, smoking, coffee consumption, and tea consumption.

**Smoking behaviour**

Smoking behaviour was self-reported with the following questions: “Do you smoke?: yes/no” (A), “Have you previously been smoking?: yes/no” (B), “Have many years have you been smoking?” (C), “How old were you when you started smoking?” (D), “If you have previously been smoking, how old were you when you stopped?” (E), “If you smoke/have been smoking, how much did you smoke on average: cigarettes without filter per day and and/or cigarettes with filter per day and/or cheroots per day and/or cigars per day and/or packs of pipe tobacco per week” (F), “Do you inhale when smoking?”(G), “Do you use nicotine replacement therapy (chewing gum, nicotine patch or the like?”: yes/no”(H), “If yes, how many years have you been using nicotine replacement therapy?”(I), “How many hours per day are you exposed to second-hand smoke?”(J). Individuals were classified as current, former, and never smokers based on A and B. If both answers were missing but participants had indicated that they smoked in either C, D, F, and/or G and had not indicated that they had stopped smoking at a certain age in E were categorised as current smokers. If both answers were missing but participants had indicated that they smoked in either C, D, F, and/or G and had indicated that they had stopped smoking at a certain age in E were categorised as former smokers. If all answers were missing participants were categorised as never smokers. Answers in F were summarised into cigarettes smoked per day or equivalent as follows: one cigarette without and/or with filter as one cigarette per day, one cheroot as 3 cigarettes per day, one cigar as 5 cigarettes per day, and one pack of pipe tobacco (50g) per week as 50/7 cigarettes per day. Cumulated smoking in pack years was calculated using the formula: pack years = cigarettes smoked per day or equivalent/20) × years smoking/smoked. Years smoking/smoked were obtained in C or as age minus D in current smokers if C was missing or as E minus D in former smokers if C was missing.

**Coffee and tea consumption**

Tea and coffee consumption were assessed using the questions “What is your average weekly consumption of coffee/tea (in cups)?”. Baseline coffee/tea intake was converted into cups/day.

**Covariates**

Age was self-reported and confirmed using the unique Danish Personal Identification Number as a continuous variable. We categorised age as <40 years, 40-50 years, 50-60 years, 60-70 years, 70-80 years, and >80 years in order to investigate linearity with tea and coffee consumption and cigarette smoking. Education since high school or equivalent (age 19) was captured as none, studying, short education (up to 3 years with books), vocational qualifications (1-3 years), college degree (≥3 years for example teacher, nurse etc.), or university degree. Participants were grouped as Low: no education/studying/vocational qualifications/short education, Middle: college degree, and High: university degree).

**References**

1. Collins R. What makes UK Biobank special? Lancet 2012;379(9822):1173-4.

2. Wain LV, Shrine N, Miller S, Jackson VE, Ntalla I, Artigas MS, et al. Novel insights into the genetics of smoking behaviour, lung function, and chronic obstructive pulmonary disease (UK BiLEVE): a genetic association study in UK Biobank. Lancet Respir Med 2015;3(10):769-81.

3. Holmen J, Midthjell K, Krüger Ø, Langhammer A, Lingaas Holmen T, Bratberg GH, et al. The Nord-Trøndelag Health Study 1995–97 (HUNT 2): objectives, contents, methods and participation. The Norwegian Journal of Epidemiology 2003;13(1):19-32.

4. Bjorngaard JH, Gunnell D, Elvestad MB, Davey Smith G, Skorpen F, Krokan H, et al. The causal role of smoking in anxiety and depression: a Mendelian randomization analysis of the HUNT study. Psychol Med 2013;43(4):711-9.

5. Zacho J, Tybjaerg-Hansen A, Jensen JS, Grande P, Sillesen H, Nordestgaard BG. Genetically elevated C-reactive protein and ischemic vascular disease. New England Journal of Medicine 2008;359(18):1897-1908.

6. Kamstrup PR, Tybjaerg-Hansen A, Steffensen R, Nordestgaard BG. Genetically Elevated Lipoprotein(a) and Increased Risk of Myocardial Infarction. Jama-Journal of the American Medical Association 2009;301(22):2331-2339.

**Table S1. Correlations between tea and coffee within studies**

|  | **All individuals drinking at least some tea or coffee** | | **Individuals drinking both tea and coffee** | |
| --- | --- | --- | --- | --- |
|  | **N** | **r** | **N** | **r** |
| **UK Biobank** | 111,582 | -0.37 | 74,729 | -0.17 |
| **HUNT** | 31,930 | -0.22 | 15,609 | 0.07 |
| **CGPS** | 77,023 | -0.35 | 38,994 | -0.08 |

r= Pearson’s correlation coefficient.

**Table S2. Association between smoking behaviour and demographic factors in UK Biobank**

|  | **N** | **Never (%)** | **Former**  **(%)** | **Current**  **(%)** | **P-value^1^** | **N** | **Difference in cigarettes smoked per day (current smokers)**  **(95% CI)** | **P-value^2^** |
| --- | --- | --- | --- | --- | --- | --- | --- | --- |
| **Total** | 114,029 | 53.7 | 34.2 | 12.1 |  |  |  |  |
| **Sex** |  |  |  |  |  |  |  |  |
| Male | 53,893 | 48.7 | 38.1 | 13.2 | <0.001 | 4,963 | REF | - |
| Female | 60,136 | 58.1 | 30.7 | 11.2 |  | 5,643 | -2.9 (-3.2, -2.6) | <0.001 |
| **Age** |  |  |  |  |  |  |  |  |
| 38-44 years | 10,350 | 58.3 | 24.4 | 17.3 | <0.001 | 1,370 | REF | - |
| 45-49 years | 14,056 | 58.7 | 25.3 | 16.1 |  | 1,781 | 1.0 (0.4, 1.6) | 0.001 |
| 50-54 years | 17,329 | 56.5 | 29.1 | 14.4 |  | 1,965 | 1.2 (0.6, 1.7) | <0.001 |
| 55-59 years | 21,083 | 54.1 | 33.9 | 12.0 |  | 1,968 | 0.8 (0.3, 1.4) | 0.003 |
| 60-64 years | 28,878 | 50.8 | 39.2 | 10.1 |  | 2,158 | 0.9 (0.4, 1.5) | 0.001 |
| 65+ years | 22,333 | 49.4 | 42.3 | 8.3 |  | 1,364 | 0.4 (-0.2, 1.0) | 0.20 |
| **Education**  None | 20,501 | 41.4 | 41.4 | 17.2 | <0.001 | 2,948 | REF | - |
| School/vocational | 40,829 | 51.9 | 34.2 | 13.9 |  | 4,507 | -0.9 (-1.3, -0.5) | <0.001 |
| Degree/professional | 51,687 | 60.0 | 31.3 | 8.7 |  | 3,036 | -1.7 (-2.1, -1.3) | <0.001 |

Data are missing for some variables.1. P-value from chi-squared test. 2. P-value from linear regression, using robust standard errors.

**Table S3. Association between smoking behaviour and demographic factors in HUNT**

|  | **N** | **Never (%)** | **Former**  **(%)** | **Current**  **(%)** | **P-value^1^** | **N** | **Difference in cigarettes smoked per day (current smokers) (95% CI)** | **P-value^2^** |
| --- | --- | --- | --- | --- | --- | --- | --- | --- |
| **Total** | 56,625 | 43.7 | 25.3 | 31.0 |  |  |  |  |
| **Sex** |  |  |  |  |  |  |  |  |
| Male | 27,009 | 37.6 | 31.8 | 30.6 | <0.001 | 7,634 | REF |  |
| Female | 29,616 | 49.2 | 19.5 | 31.3 |  | 8,957 | -2.4 (-2.5, -2.2) | <0.001 |
| **Age** |  |  |  |  |  |  |  |  |
| <40 years | 17,599 | 52.2 | 15.8 | 32.0 | <0.001 | 5,547 | REF |  |
| 40-49 years | 11,987 | 34.9 | 26.1 | 39.1 |  | 4,582 | 1.0 (0.8,1.3) | <0.001 |
| 50-59 years | 9,756 | 36.6 | 29.8 | 33.6 |  | 3,138 | 1.1 (0.9, 1.4) | <0.001 |
| 60-69 years | 7,912 | 37.6 | 33.3 | 29.1 |  | 2,049 | -0.6 (-0.8, -0.3) | <0.001 |
| 70-79 years | 6,984 | 46.8 | 33.0 | 20.2 |  | 1,113 | -2.2 (-2.5, -1.9) | <0.001 |
| 80+ years | 2,387 | 65.9 | 24.5 | 9.6 |  | 162 | -2.4 (-3.2, -1.5) | <0.001 |
| **Education** |  |  |  |  |  |  |  |  |
| Primary education (<10 years)  Primary education (<10 years) | 19,736 | 39.0 | 27.2 | 33.8 | <0.001 | 6,184 | REF |  |
| Secondary education (11-12 years) | 23,508 | 41.3 | 24.7 | 34.0 |  | 7,769 | 0.1 (-0.1, 0.3) | 0.28 |
| College/university education (>12 years) | 10,647 | 56.8 | 23.4 | 19.8 |  | 2,041 | -0.4 (-0.7, -0.1) | 0.007 |

Data are missing for some variables.1. P-value from chi-squared test. 2. P-value from linear regression, using robust standard errors.

**Table S4. Association between smoking behaviour and demographic factors in CGPS**

|  | **N** | **Never (%)** | **Former**  **(%)** | **Current**  **(%)** | **P-value^1^** | **N** | **Difference in cigarettes smoked per day (current smokers) (95% CI)** | **P-value^2^** |
| --- | --- | --- | --- | --- | --- | --- | --- | --- |
| **Total** | 78,650 | 40.90 | 40.1 | 190 |  |  |  |  |
| **Sex** |  |  |  |  |  |  |  |  |
| Male | 35,288 | 37.0 | 43.0 | 20.0 | <0.001 | 7,043 | REF | - |
| Female | 43,362 | 44.1 | 37.8 | 18.2 |  | 7,886 | -3.4 (-3.7, -3.1) | <0.001 |
| **Age** |  |  |  |  |  |  |  |  |
| <40 years | 5,604 | 57.4 | 24.3 | 18.3 | <0.001 | 1,027 | REF | - |
| 40-49 years | 18,382 | 47.5 | 33.6 | 18.8 |  | 3,459 | 1.7(1.1, 2.4) | <0.001 |
| 50-59 years | 20,027 | 36.8 | 40.8 | 22.4 |  | 4,482 | 2.3(1.7, 2.9) | <0.001 |
| 60-69 years | 20,298 | 38.9 | 42.0 | 19.1 |  | 3,879 | 2.4(1.7, 3.0) | <0.001 |
| 70-79 years | 10,875 | 34.7 | 50.0 | 15.3 |  | 1,664 | 0.30 (-0.41, 1.0) | 0.41 |
| 80+ years | 3,464 | 33.5 | 54.4 | 12.1 |  | 418 | -1.6 (-2.6, -0.55) | 0.003 |
| **Education** |  |  |  |  |  |  |  |  |
| Low | 17,560 | 38.3 | 37.0 | 24.7 | <0.001 | 4,337 | REF | - |
| Middle | 27,456 | 37.1 | 40.5 | 22.4 |  | 6,161 | -1.2 (-2.4, 0.47) | 0.52 |
| High | 33,376 | 45.4 | 41.5 | 13.1 |  | 4,369 | -2.1 (-2.5, -1.7) | <0.001 |

Data are missing for some variables. 1. P-value from chi-squared test. 2. P-value from linear regression, using robust standard errors.

**Table S5. Associations between tea and coffee consumption and demographic factors in UK Biobank**

|  | **Coffee (cups per day)** | | | **Tea (cups per day)** | | |
| --- | --- | --- | --- | --- | --- | --- |
|  | **N** | **Beta (95% CI)** | **P-value^1^** | **N** | **Beta (95% CI)** | **P-value^1^** |
| **Sex** |  |  |  |  |  |  |
| Male | 43,383 | REF | - | 46,025 | REF | - |
| Female | 46,406 | -0.21 (-0.24, -0.19) | <0.001 | 50,497 | -0.05 (-0.08, -0.01) | 0.006 |
| **Age category** |  |  |  |  |  |  |
| 38-44 years | 7,705 | REF | - | 8,312 | REF | - |
| 45-49 years | 10,639 | 0.03 (-0.03, 0.09) | 0.37 | 11,574 | 0.20 (0.13, 0.28) | <0.001 |
| 50-54 years | 13,280 | -0.01(-0.07, 0.05) | 0.85 | 14,502 | 0.29 (0.22, 0.36) | <0.001 |
| 55-59 years | 16,501 | -0.16 (-0.21, -0.10) | <0.001 | 17,891 | 0.29 (0.22, 0.36) | <0.001 |
| 60-64 years | 23,491 | -0.24 (-0.30, -0.19) | <0.001 | 24,760 | 0.20 (0.13, 0.26) | <0.001 |
| 65+ years | 18,173 | -0.34 (-0.39, -0.28) | <0.001 | 19,483 | 0.17 (0.10, 0.24) | <0.001 |
| **Education** |  |  |  |  |  |  |
| None | 14,897 | REF | - | 17,368 | REF | - |
| NVQ/HND/CSE/O-levels/A-levels | 31,667 | -0.04 (0.08, 0.001) | 0.06 | 34,106 | -0.47 (-0.52, -0.41) | <0.001 |
| Degree/professional | 42,460 | -0.18 (-0.22, -0.14) | <0.001 | 44,168 | -0.68 (-0.73, -0.63) | <0.001 |

Only includes consumers of tea and coffee in all analyses. Data are missing for some variables.

**Table S6. Associations between tea and coffee consumption and demographic factors in HUNT**

|  | **Coffee (cups per day)** | | | **Tea (cups per day)** | | |
| --- | --- | --- | --- | --- | --- | --- |
|  | **N** | **Beta (95% CI)** | **P-value^1^** | **N** | **Beta (95% CI)** | **P-value^1^** |
| **Sex** |  |  |  |  |  |  |
| Male | 24,487 | REF |  | 6,841 | REF |  |
| Female | 25,504 | -0.82 (-0.87, -0.76) | <0.001 | 11,289 | -0.10 (-0.14, -0.06) | <0.001 |
| **Age category** |  |  |  |  |  |  |
| <40 years | 13,877 | REF |  | 5,544 | REF |  |
| 40-49 years | 11,173 | 0.46 (0.37, 0.54) | <0.001 | 3,908 | 0.05 (-0.01, 0.11) | 0.08 |
| 50-59 years | 9,159 | 0.31 (0.22, 0.39) | <0.001 | 3,197 | 0.24 (0.18, 0.31) | <0.001 |
| 60-69 years | 7,340 | -0.27 (-0.36, -0.19) | <0.001 | 2,499 | 0.16 (0.10, 0.23) | <0.001 |
| 70-79 years | 6,331 | -1.01 (-1.09, -0.93) | <0.001 | 2,259 | 0.04 (-0.02, 0.10) | 0.21 |
| 80+ years | 2,111 | -1.63 (-1.73, -1.52) | <0.001 | 723 | -0.06 (-0.14, 0.02) | 0.12 |
| **Education** |  |  |  |  |  |  |
| Primary education (<10 years) | 18,259 | REF |  | 5,655 | REF |  |
| Secondary education (11-12 years) | 20,488 | 0.04 (-0.02, 0.11) | 0.164 | 7,297 | -0.10 (-0.15, -0.05) | <0.001 |
| College/university education (>12 years) | 9,073 | -0.73 (-0.80, -0.66) | <0.001 | 4,515 | -0.13 (-0.18, -0.08) | <0.001 |

Only includes consumers of tea or coffee in all analyses. Data are missing for some variables.

**Table S7. Associations between tea and coffee consumption and demographic factors in CGPS**

|  | **Coffee (cups per day)** | | | **Tea (cups per day)** | | |
| --- | --- | --- | --- | --- | --- | --- |
|  | **N** | **Beta (95% CI)** | **P-value^1^** | **N** | **Beta (95% CI)** | **P-value^1^** |
| **Sex** |  |  |  |  |  |  |
| Male | 32,431 | REF | - | 17,078 | REF | - |
| Female | 37,797 | -0.67 (-0.70, -0.63) | <0.001 | 28,711 | 0.24 (0.20, 0.27) | <0.001 |
| **Age category** |  |  |  |  |  |  |
| <40 years | 3,935 | REF |  | 3,343 | REF |  |
| 40-49 years | 15,684 | 0.77 (0.70, 0.84) | <0.001 | 11,089 | 0.65 (0.58, 0.72) | <0.001 |
| 50-59 years | 18,281 | 0.78 (0.71, 0.86) | <0.001 | 11,648 | 0.75 (0.68, 0.82) | <0.001 |
| 60-69 years | 19,020 | 0.38 (0.31, 0.45) | <0.001 | 11,454 | 0.62 (0.55, 0.69) | <0.001 |
| 70-79 years | 10,160 | -0.04 (-0.11, 0.04) | 0.352 | 6,166 | 0.54 (0.46, 0.61) | <0.001 |
| 80+ years | 3,148 | -0.45 (-0.55, -0.35) | <0.001 | 2,089 | 0.52 (0.42, 0.62) | <0.001 |
| **Education** |  |  |  |  |  |  |
| None | 15,382 | REF | - | 9,186 | REF | - |
| 3 years or less higher education | 24,827 | 0.15 (0.10, 0.19) | <0.001 | 13,880 | -0.01 (-0.06, 0.04) | 0.643 |
| More than 3 years higher education | 29,783 | -0.07 (-0.11,-0.03) | 0.001 | 22,589 | 0.26 (0.22, 0.31) | <0.001 |

Only includes consumers of tea or coffee in all analyses. Data are missing for some variables.

**Table S8. Associations between smoking status and tea and coffee consumption**

|  |  | **Age, sex, adjusted** | **Age, sex, education, adjusted** | **Age, sex, education, tea/coffee adjusted** |  |
| --- | --- | --- | --- | --- | --- |
|  | **N** | **Beta (95% CI)** | **Beta (95% CI)** | **Beta (95% CI)** | |
| **UK Biobank** |  |  |  |  | |
| **Coffee (cups per day)** |  |  |  |  | |
| Never smokers | 47,333 | REF | REF | REF | |
| Former smokers | 30,844 | 0.27 (0.24, 0.30) | 0.26 (0.23, 0.29) | 0.24 (0.21, 0.26) | |
| Current smokers | 10,847 | 1.21 (1.16, 1.27) | 1.19 (1.14, 1.25) | 1.21 (1.16. 1.26) | |
| **Tea (cups per day)** |  |  |  |  | |
| Never smokers | 51,946 | REF | REF | REF | |
| Former smokers | 32,711 | 0.08 (0.05, 0.12) | 0.04 (0.006, 0.08) | 0.10 (0.06, 0.13) | |
| Current smokers | 10,985 | 0.87 (0.80, 0.93) | 0.77 (0.70, 0.84) | 1.02 (0.95, 1.09) | |
| **HUNT** |  |  |  |  | |
| **Coffee (cups per day)** |  |  |  |  | |
| Never smokers | 20,129 | 19,477 | REF | REF^1^ | |
| Former smokers | 12,943 | 12,677 | 0.95 (0.89, 1.01) | 0.93 (0.87, 0.99) | |
| Current smokers | 16,026 | 15,634 | 2.42 (2.36, 2.49) | 2.33 (2.27, 2.40) | |
| **Tea (cups per day)** |  |  |  |  | |
| Never smokers | 9,683 | REF | REF | REF^2^ | |
| Former smokers | 4,428 | 0.15 (0.10, 0.20) | 0.15 (0.10, 0.20) | 0.16 (0.12, 0.21) | |
| Current smokers | 3,346 | 0.21 (0.15, 0.27) | 0.20 (0.14, 0.26) | 0.24 (0.18, 0.31) | |
| **CPGS** |  |  |  |  | |
| **Coffee (cups per day)** |  |  |  |  | |
| Never smokers | 27,264 | REF | REF | REF | |
| Former smokers | 29,110 | 0.24 (0.21, 0.27) | 0.24 (0.21, 0.27) | 0.22 (0.19, 0.25) | |
| Current smokers | 13,854 | 1.06 (1.01, 1.11) | 1.02 (0.98, 1.07) | 0.95 (0.90, 1.00) | |
| **Tea (cups per day)** |  |  |  |  | |
| Never smokers | 21,273 | REF | REF | REF | |
| Former smokers | 18,499 | -0.08 (-0.11, -0.05) | -0.08 (-0.12, -0.05) | -0.03 (-0.07, 0.01) | |
| Current smokers | 6,017 | -0.15 (-0.20, -0.09) | -0.10 (-0.15, -0.04) | 0.04 (-0.02, 0.09) | |

Beta coefficients represent difference in coffee/tea consumption in former and current compared to never smokers. From linear regression using robust standard errors to account for non- normality of residuals.

^1^N reduced to 26,328 due to missing data on tea consumption.

^2^N reduced to 16,895 due to missing data on coffee consumption.

**Table S9. Associations between number of cigarettes per day among current smokers and coffee and tea consumption**

|  | **N** | **Age, sex adjusted** | | **Age, sex, education, adjusted** | | **Age, sex, education, tea/coffee adjusted** | |
| --- | --- | --- | --- | --- | --- | --- | --- |
|  |  | **Beta (95% CI)** | **P-value** | **Beta (95% CI)** | **P-value** | **Beta (95% CI)** | **P-value** |
| **UK Biobank** |  |  |  |  |  |  |  |
| Coffee (cups per day) | 8,163 | 0.058 (0.049, 0.067) | <0.001 | 0.058 (0.049, 0.067) | <0.001 | 0.064 (0.056, 0.072) | <0.001 |
| Tea per day (cups per day) | 8,258 | 0.071 (0.059, 0.082) | <0.001 | 0.067 (0.055, 0.078) | <0.001 | 0.077 (0.066, 0.087) | <0.001 |
| **HUNT** |  |  |  |  |  |  |  |
| Coffee (cups per day) | 14,948 | 0.195 (0.181, 0.208) | <0.001 | 0.192 (0.178, 0.205) | <0.001 | 0.186^1^ (0.165, 0.207) | <0.001 |
| Tea per day (cups per day) | 3,207 | 0.031 (0.017, 0.046) | <0.001 | 0.030 (0.015, 0.045) | <0.001 | 0.033^2^ (0.017, 0.049) | <0.001 |
| **CGPS** |  |  |  |  |  |  |  |
| Coffee (cups per day) | 13,854 | 0.053 (0.047, 0.059) | <0.001 | 0.052 (0.045, 0.0578) | <0.001 | 0.050 (0.044, 0.056) | <0.001 |
| Tea per day (cups per day) | 6,017 | 0.007 (0.001, 0.013) | 0.018 | 0.009 (0.003, 0.015) | 0.003 | 0.013 (0.007, 0.019) | <0.001 |

Beta coefficients represent change in coffee and tea per additional cigarette smoked per day

^1^N reduced to 6,511 due to missing data on tea consumption.

^2^N reduced to 3,124 due to missing data on coffee consumption.

**Table S10. Associations of rs16969968/rs1051730 genotype with smoking heaviness**

|  | **N** | **Major homozygotes** | **Heterozygotes** | **Minor homozygotes** | **Beta (95% CI)^1^** | **P-value** |
| --- | --- | --- | --- | --- | --- | --- |
|  |  | **Mean (SD)** | **Mean (SD)** | **Mean (SD)** |  |  |
| **UK Biobank** |  |  |  |  |  |  |
| **Cigarettes per day (current smokers)** | 10,606 | N=4,469  16.4 (8.0) | N=4,867  17.2 (8.3) | N=1,270  18.3 (8.3) | 0.94 (0.72, 1.17) | <0.001 |
| **Pack years**  **(ever smokers)** | 40,343 | N= 17,296  26.2 (18.1) | N=18,402  27.9 (19.3) | N= 4,645  29.1 (19.6) | 1.64 (1.37, 1.91) | <0.001 |
| **HUNT** |  |  |  |  |  |  |
| **Cigarettes per day (current smokers)** | 16,591 | N=7,054  10.7 (5.6) | N=7,510  11.4 (5.7) | N=2,027  12.1 (5.9) | 0.67 (0.54, 0.79) | <0.001 |
| **Pack years**  **(ever smokers)** | 28,697 | N=12,597  13.0 (11.3) | N=12,787  14.0 (11.6) | N= 3,313  14.8 (12.0) | 1.04 (0.87, 1.22) | <0.001 |
| **CGPS** |  |  |  |  |  |  |
| **Cigarettes per day (current smokers)** | 14,929 | N=6,525  14.8 (9.2) | N=6,757  15.6 (8.9) | N=1,647  16.8(9.6) | 0.94 (0.72, 1.16) | <0.001 |
| **Pack years**  **(ever smokers)** | 46,482 | N=21,108  20.6 (19.7) | N=20,518  22.2 (20.3) | N=4,856  23.7 (21.5) | 1.67 (1. 40, 1.94) | <0.001 |

1. Coefficients from linear regression, per additional copy of the minor allele. Adjusted for age and sex in all studies and principal components in UK Biobank.

**Table S11. Association of rs16969968 genotype with demographic factors in all smoking categories combined in UK Biobank**

|  | **rs169699968 genotype** | | | |
| --- | --- | --- | --- | --- |
|  | **GG** | **GA** | **AA** | **P-value^1^** |
|  | **N (%)** | **N (%)** | **N (%)** |  |
| **Sex** |  |  |  |  |
| Male | 23,927 (44.4) | 24,103 (44.7) | 5,863 (10.9) | 0.40 |
| Female | 26,509 (44.1) | 26,958 (44.8) | 6,669 (11.1) |  |
| **Education** |  |  |  |  |
| None | 9,101 (44.4) | 9,264 (45.2) | 2,136 (10.5) | 0.03 |
| School/vocational | 17,972 (44.0) | 18,250 (44.7) | 4,607 (11.3) |  |
| Degree/professional | 22,925 (44.4) | 23,078 (44.7) | 5,684 (11.0) |  |
|  | **Mean (SD)** | **Mean (SD** | **Mean (SD** |  |
| **Age in years** | 56.9 (7.9) | 56.9 (7.9) | 56.7 (7.9) | 0.05 |

Data are missing for some variables.

1. P-value from chi-squared tests for categorical variables and from linear regression for age.

**Table S12. Association of rs16969968 genotype with demographic factors in never smokers in UK Biobank**

|  | **rs169699968 genotype** | | |  |
| --- | --- | --- | --- | --- |
|  | **GG** | **GA** | **AA** | **P-value^1^** |
|  | **N (%)** | **N (%)** | **N (%)** |  |
| **Sex** |  |  |  |  |
| Male | 11,734 (44.7) | 11,642 (44.3) | 2,878 (11.0) | 0.38 |
| Female | 15,437 (44.2) | 15,682 (44.9) | 3,815 (10.9) |  |
| **Education** |  |  |  |  |
| None | 3,728 (43.9) | 3,873 (45.6) | 894 (10.6) | 0.16 |
| School/vocational | 9,403 (44.4) | 9,406 (44.4) | 2,381 (11.2) |  |
| Degree/professional | 13,836 (44.6) | 13,808 (44.5) | 3,349 (10.8) |  |
|  | **Mean (SD)** | **Mean (SD** | **Mean (SD** |  |
| **Age in years** | 56.3 (8.0) | 56.5 (8.0) | 56.3 (8.0) | 0.50 |

Data are missing for some variables.

1. P-value from chi-squared tests for categorical variables and from linear regression for age.

**Table S13. Association of rs16969968 genotype with demographic factors in former smokers in UK Biobank**

|  | **rs169699968 genotype** | | |  |
| --- | --- | --- | --- | --- |
|  | **GG** | **GA** | **AA** | **P-value^1^** |
|  | **N (%)** | **N (%)** | **N (%)** |  |
| **Sex** |  |  |  |  |
| Male | 9,091 (44.2) | 9,254 (45.0) | 2,206 (10.7) | 0.28 |
| Female | 8,144 (44.1) | 8,238 (44.6) | 2,073 (11.2) |  |
| **Education** |  |  |  |  |
| None | 3,824 (45.1) | 3,791 (44.7) | 867 (10.2) | 0.03 |
| School/vocational | 6,089 (43.6) | 6,336 (45.3) | 1,542 (11.0) |  |
| Degree/professional | 7,138 (44.1) | 7,194 (44.5) | 1,842 (11.4) |  |
|  | **Mean (SD)** | **Mean (SD)** | **Mean (SD)** |  |
| **Age in years** | 58.5 (7.5) | 58.4 (7.6) | 58.1 (7.6) | 0.002 |

Data are missing for some variables.

1. P-value from chi-squared tests for categorical variables and from linear regression for age.

**Table S14. Association of rs16969968 genotype with demographic factors in current smokers in UK Biobank**

|  | **rs169699968 genotype** | | |  |
| --- | --- | --- | --- | --- |
|  | **GG** | **GA** | **AA** | **P-value^1^** |
|  | **N (%)** | **N (%)** | **N (%)** |  |
| **Sex** |  |  |  |  |
| Male | 3,102 (43.8) | 3,207 (45.3) | 779 (11.0) | 0.43 |
| Female | 2,928 (43.3) | 3,038 (45.0) | 790 (11.7) |  |
| **Education** |  |  |  |  |
| None | 1,549 (44.0) | 1,600 (45.4) | 375 (10.7) | 0.14 |
| School/vocational | 2,471 (43.6) | 2,508 (44.3) | 684 (12.1) |  |
| Degree/professional | 1,951 (43.2) | 2,076 (46.0) | 493 (10.9) |  |
|  | **Mean (SD)** | **Mean (SD)** | **Mean (SD)** |  |
| **Age in years** | 55.0 (8.0) | 54.9 (8.0) | 54.6 (8.0) | 0.08 |

Data are missing for some variables.

1. P-value from chi-squared tests for categorical variables and from linear regression for age.

**Table S15. Association of rs1051730 genotype with demographic factors in all smoking categories combined in HUNT**

|  | **rs1051730 genotype** | | | |
| --- | --- | --- | --- | --- |
|  | **CC** | **CT** | **TT** | **P-value^1^** |
|  | **N (%)** | **N (%)** | **N (%)** |  |
| **Sex** |  |  |  |  |
| Male | 12,043 (44.6) | 11,954 (44.2) | 3,024 (11.2) | 0.55 |
| Female | 13,100 (44.2) | 13,157 (44.4) | 3,386(11.4) |  |
| **Education** |  |  |  |  |
| Primary education (<10 years) | 8,668 (43.9) | 8,850 (44.8) | 2,241 (11.3) | 0.22 |
| Secondary education (11-12 years) | 10,487 (44.6) | 10,389 (44.2) | 2,638 (11.2) |  |
| College/university education (>12 years) | 4,800 (45.1) | 4,631 (43.5) | 1,222 (11.5) |  |
|  | **Mean (SD)** | **Mean (SD)** | **Mean (SD)** |  |
| **Age in years** | 50.0 (17.1) | 49.9 (17.1) | 49.5 (17.0) | 0.07 |

Data are missing for some variables.

1. P-value from chi-squared tests for categorical variables and from linear regression for age.

**Table S16. Association of rs1051730 genotype with demographic factors in never smokers in HUNT**

|  | **rs1051730 genotype** | | |  |
| --- | --- | --- | --- | --- |
|  | **CC** | **CT** | **TT** | **P-value^1^** |
|  | **N (%)** | **N (%)** | **N (%)** |  |
| **Sex** |  |  |  |  |
| Male | 4,558 (44.9) | 4,483 (44.1) | 1,122 (11.0) | 0.87 |
| Female | 6,580 (45.1) | 6,383 (43.8) | 1,621 (11.1) |  |
| **Education** |  |  |  |  |
| Primary education (<10 years) | 3,447 (44.8) | 3,424 (44.5) | 832 (10.8) | 0.72 |
| Secondary education (11-12 years) | 4,389 (45.3) | 4,230 (43.6) | 1,079 (11.1) |  |
| College/university education (>12 years) | 2,742 (45.3) | 2,626 (43.4) | 681 (11.3) |  |
|  | **Mean (SD)** | **Mean (SD)** | **Mean (SD)** |  |
| **Age in years** | 49.2 (18.8) | 49.3 (18.8) | 49.1 (18.9) | 0.98 |

Data are missing for some variables.

1. P-value from chi-squared tests for categorical variables and from linear regression for age.

**Table S17. Association of rs1051730 genotype with demographic factors in former smokers in HUNT**

|  | **rs1051730 genotype** | | |  |
| --- | --- | --- | --- | --- |
|  | **CC** | **CT** | **TT** | **P-value^1^** |
|  | **N (%)** | **N (%)** | **N (%)** |  |
| **Sex** |  |  |  |  |
| Male | 3,951 (46.1) | 3,744 (43.7) | 881 (10.3) | 0.12 |
| Female | 2,586 (44.8) | 2,540 (44.0) | 648 (11.2) |  |
| **Education** |  |  |  |  |
| Primary education (<10 years) | 2,411 (45.0) | 2,353 (43.9) | 594 (11.1) | 0.26 |
| Secondary education (11-12 years) | 2,707 (46.6) | 5,509 (43.2) | 591 (10.2) |  |
| College/university education (>12 years) | 1,114 (44.8) | 1,098 (44.1) | 277 (11.1) |  |
|  | **Mean (SD)** | **Mean (SD)** | **Mean (SD)** |  |
| **Age in years** | 54.3 (15.6) | 54.0 (15.5) | 53.9 (15.5) | 0.27 |

Data are missing for some variables.

1. P-value from chi-squared tests for categorical variables and from linear regression for age.

**Table S18. Association of rs1051730 genotype with demographic factors in current smokers in HUNT**

|  | **rs1051730 genotype** | | |  |
| --- | --- | --- | --- | --- |
|  | **CC** | **CT** | **TT** | **P-value^1^** |
|  | **N (%)** | **N (%)** | **N (%)** |  |
| **Sex** |  |  |  |  |
| Male | 3,530 (42.7) | 3,720 (45.0) | 1,020 (12.3) | 0.68 |
| Female | 3,920 (42.3) | 4,222 (45.6) | 1,116 (12.1) |  |
| **Education** |  |  |  |  |
| Primary education (<10 years) | 2,801 (42.0) | 3,061 (45.9) | 813 (12.2) | 0.19 |
| Secondary education (11-12 years) | 3,387 (42.3) | 3,648 (45.6) | 968 (12.1) |  |
| College/university education (>12 years) | 940 (44.6) | 905 (42.9) | 264 (12.5) |  |
|  | **Mean (SD)** | **Mean (SD)** | **Mean (SD)** |  |
| **Age in years** | 47.5 (15.0) | 47.5 (14.9) | 46.9 (14.5) | 0.28 |

Data are missing for some variables. 1. P-value from chi-squared tests for categorical variables and from linear regression for age.

**Table S19. Association of rs1051730 genotype with demographic factors in all smoking categories combined in CGPS**

|  | **rs1051730 genotype** | | | |
| --- | --- | --- | --- | --- |
|  | **CC** | **CT** | **TT** | **P-value^1^** |
|  | **N (%)** | **N (%)** | **N (%)** |  |
| **Sex** |  |  |  |  |
| Male | 15,921 (45.1) | 15,613 (44.2) | 3,754 (10.7) | 1.00 |
| Female | 19,565 (45.1) | 19,178 (44.2) | 4,619 (10.6) |  |
| **Education** |  |  |  |  |
| None | 7,905 (45.0) | 7,809 (44.5) | 1,846 (10.5) | 0.77 |
| 3 years or less higher education | 12,333 (44.9) | 12,211 (44.5) | 2,912 (10.6) |  |
| More than 3 years higher education | 15,126 (45.3) | 14,664 (43.9) | 3,586 (10.7) |  |
|  | **Mean (SD)** | **Mean (SD)** | **Mean (SD)** |  |
| **Age in years** | 57.7 (13.1) | 57.7 (13.0) | 57.3 (13.0) | 0.07 |

Data are missing for some variables.

1. P-value from chi-squared tests for categorical variables and from linear regression for age.

**Table S20. Association of rs1051730 genotype with demographic factors in never smokers in CGPS**

|  | **rs1051730 genotype** | | | |
| --- | --- | --- | --- | --- |
|  | **GG** | **GA** | **AA** | **P-value^1^** |
|  | **N (%)** | **N (%)** | **N (%)** |  |
| **Sex** |  |  |  |  |
| Male | 5.873 (45.0) | 5.752 (44.0) | 1.436 (11.0) | 0.61 |
| Female | 8.505 (44.5) | 8.521 (44.6) | 2.081 (10.9) |  |
| **Education** |  |  |  |  |
| None | 2,949 (43.8)) | 3,079 (45.7) | 703 (10.5) | 0.05 |
| 3 years or less higher education | 4,515 (44.3) | 4,545 (44.7)) | 1,117 (11.0) |  |
| More than 3 years higher education | 6,864 (45.26) | 6,616 (43.62) | 1,686 (11.1) |  |
|  | **Mean (SD)** | **Mean (SD)** | **Mean (SD)** |  |
| **Age in years** | 55.6 (13.1) | 56.0 (13.0) | 55.6 (13.0) | 0.39 |

Data are missing for some variables.

1. P-value from chi-squared tests for categorical variables and from linear regression for age.

**Table S21. Association of rs1051730 genotype with demographic factors in former smokers in CGPS**

|  | **rs1051730 genotype** | | | |
| --- | --- | --- | --- | --- |
|  | **GG** | **GA** | **AA** | **P-value^1^** |
|  | **N (%)** | **N (%)** | **N (%)** |  |
| **Sex** |  |  |  |  |
| Male | 7,003 (46.1) | 6,643 (43.8) | 1,538 (10.2) | 0.89 |
| Female | 7,580 (46.3) | 7,118 (43.5) | 1,671 (10.1) |  |
| **Education** |  |  |  |  |
| None | 3,062 (47.2)) | 2,768 (42.6) | 661 (10.2) | 0.60 |
| 3 years or less higher education | 5,113 (46.0) | 4,892 (44.0) | 1,113 (10.0) |  |
| More than 3 years higher education | 6,358 (45.9) | 6,060 (43.8) | 1,423 (10.3) |  |
|  | **Mean (SD)** | **Mean (SD)** | **Mean (SD)** |  |
| **Age in years** | 60.1 (12.7) | 60.0 (12.6) | 59.6 (12.7) | 0.08 |

Data are missing for some variables.

1. P-value from chi-squared tests for categorical variables and from linear regression for age.

**Table S22. Association of rs1051730 genotype with demographic factors in current smokers in CGPS**

|  | **rs1051730 genotype** | | | |
| --- | --- | --- | --- | --- |
|  | **GG** | **GA** | **AA** | **P-value^1^** |
|  | **N (%)** | **N (%)** | **N (%)** |  |
| **Sex** |  |  |  |  |
| Male | 3,045 (43.2) | 3,218 (45.7) | 780 (11.1) | 0.54 |
| Female | 3,480 (44.1) | 3,539 (44.9) | 867 (11.0) |  |
| **Education** |  |  |  |  |
| None | 1,893 (43.7) | 1,962 (45.2)) | 482 (11.1)) | 0.93 |
| 3 years or less higher education | 2,705 (43.9) | 2,774 (45.0) | 682 (11.1) |  |
| More than 3 years higher education | 1,904 (43.6) | 1,988 (45.5) | 477 (10.9) |  |
|  | **Mean (SD)** | **Mean (SD)** | **Mean (SD)** |  |
| **Age in years** | 56.8 (12.2) | 56.5 (12.3) | 56.6 (11.9) | 0.21 |

Data are missing for some variables.

1. P-value from chi-squared tests for categorical variables and from linear regression for age

**Table S23. Association of coffee genetic risk score with coffee consumption in UK Biobank and CPGS**

|  | **N** | **Beta (95% CI)** | **P-value** |
| --- | --- | --- | --- |
| **Current daily smokers** |  |  |  |
| UK Biobank | 10,665 | 0.07 (0.01, 0.14) | 0.02 |
| CPGS | 14,929 | 0.10 (0.07,0.13) | <0.001 |
| Combined estimate | 25,594 | 0.09 (0.07, 0.12) | <0.001 |
| **Current daily smokers (coffee consumers only)** |  |  |  |
| UK Biobank | 8,285 | 0.09 (0.03, 0.16) | 0.005 |
| CPGS | 13,854 | 0.10 (0.07,0.12) | <0.001 |
| Combined estimate | 22,139 | 0.10 (0.08, 0.12) | <0.001 |

Adjusted for age and sex (in both studies) and genetic principal components in UK Biobank. Beta coefficients are cups of coffee per day per additional coffee consumption increasing allele of the coffee genetic risk score.

**Table S24. Association of coffee genetic risk score with daily cigarette consumption in UK Biobank and CPGS**

|  | **N** | **Beta (95% CI)** | **P-value** |
| --- | --- | --- | --- |
| **Full sample** |  |  |  |
| UK Biobank | 10,665 | 0.03 (-0.14, 0.19) | 0.76 |
| CPGS | 14,929 | -0.04 (-0.12,0.04) | 0.35 |
| Combined estimate | 25,594 | -0.01 (-0.07, 0.05) | 0.77 |
| **Coffee consumers only** |  |  |  |
| UK Biobank | 8,285 | 0.003 (-0.18, 0.19) | 0.98 |
| CPGS | 13,854 | -0.04 (-0.13, 0.04) | 0.36 |
| Combined estimate | 22,139 | -0.03 (-0.11, 0.05) | 0.41 |
| **Non-consumers only** |  |  |  |
| UK Biobank | 2,380 | 0.07 (-0.29, 0.44) | 0.69 |
| CPGS | 1,075 | 0.02 (-0.28,0.33) | 0.90 |
| Combined estimate | 3,455 | 0.04 (-0.19, 0.28) | 0.73 |

Adjusted for age and sex (in both studies) and genetic principal components in UK Biobank. Beta coefficients are cigarettes per day per additional coffee consumption increasing allele of the coffee genetic risk score.

**Figure S1. Flowchart of UK Biobank study participants**

**
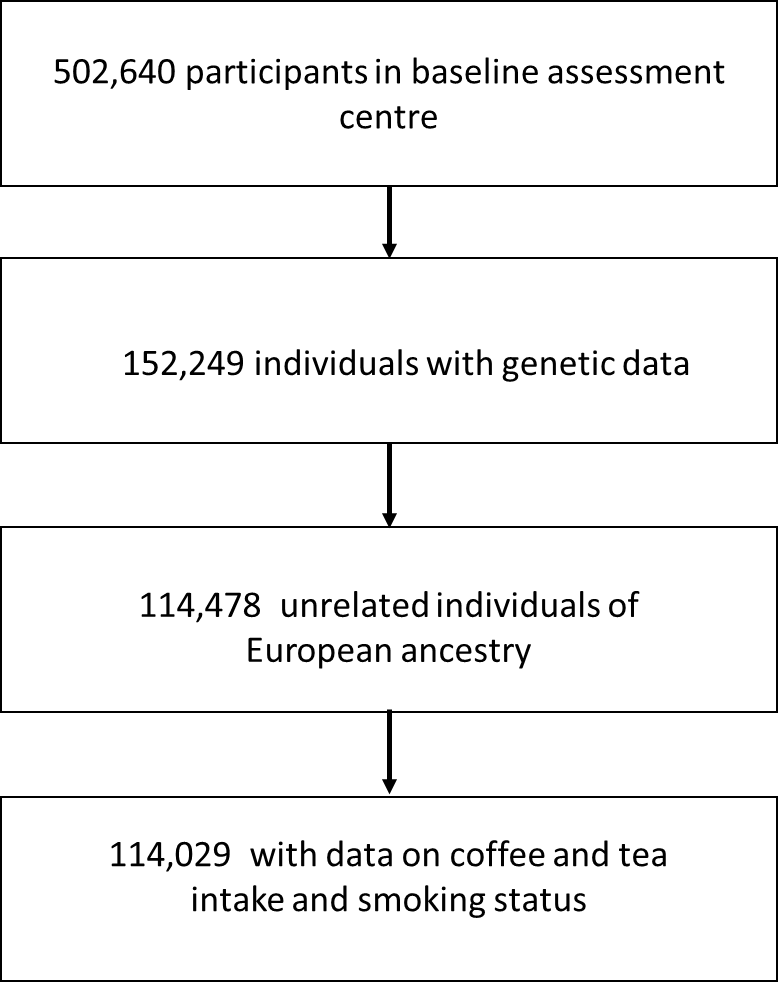
**

**Figure S2. Flowchart of HUNT study participants**

**
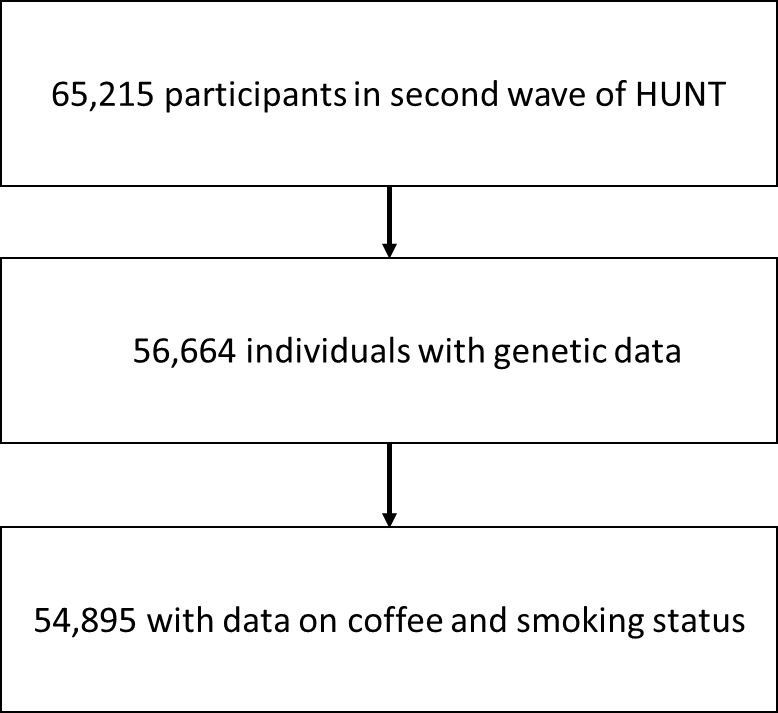
**

**Figure S3. Flowchart of CGPS study participants**

**
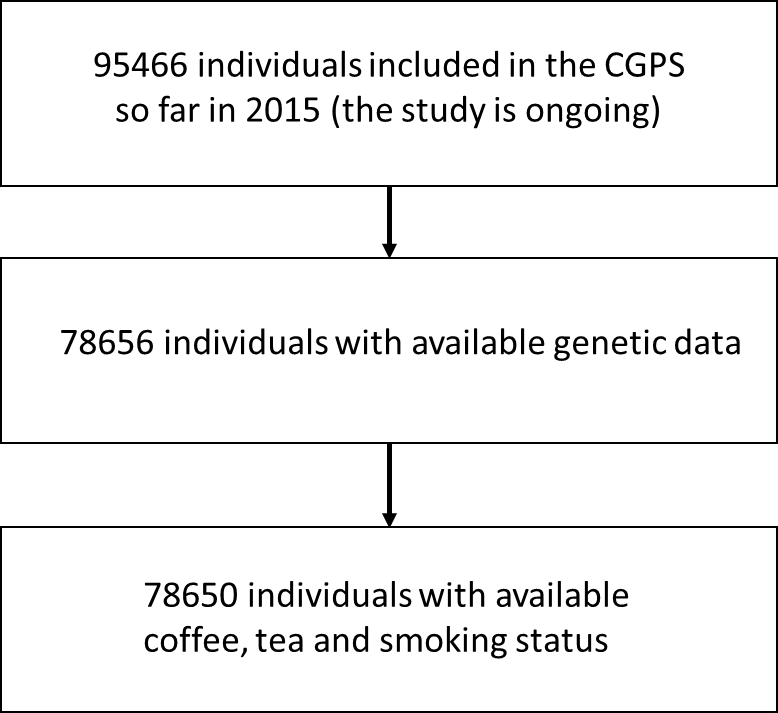
**

**Supplementary Figure S4. Associations between rs16969968/rs1051730 and consumption of any coffee**


**Supplementary Figure S5. Associations between rs16969968/rs1051730 and consumption of any coffee or tea**

**Supplementary Figure S6. Associations between rs16969968/rs1051730 and consumption of any tea**

Adjusted for age, sex (in all studies) and principal components (in UK Biobank).

**Supplementary Figure S7. Associations between rs16969968/rs1051730 and coffee consumption including non-consumers**

Adjusted for age, sex (in all studies) and principal components (in UK Biobank).

**Supplementary Figure S8. Associations between rs16969968/rs1051730 and tea consumption including non-consumers**

Adjusted for age, sex (in all studies) and principal components (in UK Biobank).

**Supplementary Figure S9. Associations between rs16969968/rs1051730 and combined coffee and tea consumption**

****Adjusted for age, sex (in all studies) and principal components (in UK Biobank). Analyses restricted to individuals reporting at least some coffee or tea consumption.

**Supplementary Figure S10. Associations between rs16969968/rs1051730 and ratio of coffee to tea**

Adjusted for age, sex (in all studies) and principal components (in UK Biobank). Analyses restricted to individuals reporting both some coffee and tea consumption.

**Supplementary Figure S11. Associations between rs16969968/rs1051730 and preference for coffee**

Adjusted for age, sex (in all studies) and principal components (in UK Biobank). Analyses restricted to individuals reporting some coffee or tea consumption. Individuals drinking more coffee than tea coded as 1, individuals drinking more tea than coffee coded as 0. Individuals consuming equal amounts of tea and coffee excluded from analyses.

**Supplementary Figure S12. Associations between rs16969968 and consumption of caffeinated and decaffeinated coffee in UK Biobank**

**Supplementary Figure S13. Association between rs16969968 and water consumption in UK Biobank**

Water consumption was assessed via a single question in the touchscreen questionnaire “How many glasses of water do you drink on average per day?”. Responses were on a continuous scale.

**Supplementary Figure S14. Association between rs16969968 and coffee or tea excluding the UKBileve sample in UK Biobank**
